# More science friction for less science fiction

**DOI:** 10.1371/journal.pbio.3003167

**Published:** 2025-05-09

**Authors:** Jennifer A. Byrne, Stefan Stender

**Affiliations:** 1 NSW Health Statewide Biobank, NSW Health Pathology, Camperdown, Australia; 2 School of Medical Sciences, Faculty of Medicine and Health, The University of Sydney, Camperdown, Australia; 3 Department of Clinical Biochemistry, Rigshospitalet, Copenhagen University Hospital, Copenhagen, Denmark; 4 Faculty of Health and Medical Sciences, University of Copenhagen, Copenhagen, Denmark

## Abstract

A new study by Suchak et al. describes a sudden, recent explosion in papers analyzing the NHANES health dataset, where similar manuscripts have been described as overwhelming processes at some journals. In this accompanying Primer article, we summarize the study's findings and recommendations. We outline practical steps that publishers, journals, peer reviewers and readers can take to better recognize similarly formulaic, low-value submissions and publications to reduce their research impacts.

## Teaser

AI-ready health datasets can be exploited to generate many research articles with potentially limited scientific value. A study in PLOS Biology highlights this problem, by describing a recent, sudden explosion in papers analyzing the NHANES health dataset.

Science fiction novels are appreciated by many readers worldwide. Scientists in particular can enjoy exploring imaginary worlds, unhindered by concerns about whether described events took place, or could add to our understanding of life as we know it. At the same time, most scientists expect descriptions of unlikely or impossible scenarios to be restricted to novels. A new study in PLOS Biology provides evidence that science fiction may also be found in the literature, by quantifying a recent, sudden explosion in papers that describe superficial and at times incomplete analyses of the National Health and Nutrition Examination Survey (NHANES) dataset [1]. Suchak and colleagues propose that the recent availability of population-level, artificial intelligence- (AI) ready health datasets that encompass many individual variables could provide opportunities to scale low-value manuscript production by individual authors and paper mills. They then propose several recommendations to address this problem.

The paper by Suchak and colleagues adds to a growing body of literature describing sudden increases in repetitive papers that describe superficial or cursory analyses of complex systems. An explosion in the numbers of published meta-analyses was described in 2013 by Ioannidis and colleagues [2]. These authors noted frequent meta-analyses that used only literature-based data and focused on 1–2 genes or variants that were typically identified through candidate gene approaches. Other authors recognized that meta-analyses might represent a valued approach to achieve publications in settings where authors have limited time or resources to undertake research [3].

The very rapid increase in publications described by Suchak and colleagues reflects other recent descriptions of rising numbers of formulaic manuscripts and publications that analyze population-level health datasets [4–8]. Rapid influxes of low-value manuscripts have been described as overwhelming some journals and peer reviewers [5,8] and reducing journal capacity to prioritize other activities that would benefit their readers [7]. Explosive increases in manuscripts and publications reporting superficial two-sample Mendelian randomization analyses were described in 2024, from the perspectives of journal editors [6] and peer reviewers [5]. These increases were proposed to be enabled by accessible, easy-to-use data analysis pipelines. Large language models could then draft the resulting manuscript, allowing the research process to be as “frictionless” as possible. The generation of formulaic manuscripts by both individuals and paper mills could underpin the marked increases in manuscripts and publications describing superficial and at times implausible analyses of population health datasets.

The study by Suchak and colleagues adds important detail and metrics to this problem. Using a systematic literature search, the authors identified 341 NHANES-based research papers published over the past decade, up to October 2024. The search was confined to single-factor analyses, i.e., studies testing associations between a predictor and a health condition in the NHANES dataset. The number of papers identified averaged four per year between 2014 and 2021, followed by a surge resembling an exponential growth pattern, with 33, 82, and 190 published in 2022, 2023, and 2024, respectively. Shortcomings of these single-factor papers included the failure to recognize or consider the likelihood of multi-factorial relationships and the risks of false discoveries. Importantly, detailed analyses identified many papers that restricted NHANES data analyses to limited date ranges or cohort subsets without explicit justifications, potentially signaling “data dredging” and/or HARKing (i.e., Hypothesizing After Results are Known).

Given recent descriptions of journal editors rejecting repetitive NHANES-based manuscripts [4,7,8], it is important to recognize that Suchak and colleagues studied publications that had, by definition, passed editorial screening and peer review. Reanalysis of the statistical associations in a subset of 28 papers that focused on depression found 13 examples where associations remained statistically significant after false discovery rate correction, and Suchak and colleagues recognized that some of these papers could have scientific value. At the same time, with so many NHANES-derived manuscripts being received by some journals, editors and peer reviewers need look beyond statistical significance, to consider whether underlying hypotheses and findings are plausible and contextually relevant [8].

Recognizing the possible impacts of scaled misuse of AI-assisted workflows, the authors provide several recommendations to address this problem, which add to or reinforce recommendations made elsewhere (Table 1). Low-value manuscripts clearly benefit from “frictionless” publication and review processes [9], and numerous recommendations in Table 1 describe more stringent manuscript requirements. However, authors and paper mills can adapt to changed manuscript requirements, particularly where these requirements are not rigorously enforced.

**Table 1:** Summary of recommendations to reduce low-value manuscripts and publications based on health datasets.

Approach	Stakeholder(s)	Action	Reference(s)
**Incentives**	Funders, institutions	Remove incentives that prioritize/reward publications over knowledge generation	[6,9]
**Information sharing**	Journals, publishers	Share intelligence around unexpected spikes in repetitive/low-value submissions	[4]
**Data access**	Data providers	Provide API keys and unique application identifiers to all dataset users	[1]
**Manuscript requirements**	Journals, publishers	Targeted study pre-registration requirements	[9]
		Adherence to reporting guidelines, e.g., STROBE-MR	[6,8]
		Analysis of full datasets unless explicitly justified	[1]
		False discovery rate corrections of statistics results	
		Discussion of contextual relevance	[8]
		Disclosure of data access/project identifiers	[1]
		Institutional email address for at least one author	[7]
**Peer review**	Journals	Desk rejection of low-value manuscripts	[1,5,6]
	Journals, publishers	Dedicated in-house statistics reviewers	[1]
		Customizable manuscript rejection templates	[1,5]
**Post-publication commentary and correction**	Readers, journals	Post-publication descriptions of problematic research	[1]
	Journals, publishers	Timely publication of expressions of concern/retractions	

It is therefore important that recommendations target other aspects of the publication process, in addition to manuscript requirements (Table 1). Where manuscripts require minimal time and resources to produce, peer review is likely to represent the rate-limiting step for publication [9]. Other recommendations therefore involve applying targeted “friction” during peer review, for example by supplying customizable rejection templates that enable scaled manuscript rejection. It will be important to ensure that such changes specifically disadvantage low-value submissions, and do not inadvertently disadvantage quality science [9].

Unfortunately, in publish-or-perish environments, investments in population-level health datasets that are intended to enable quality research can simply represent a means to produce publications with limited or no scientific value. Once the use of a particular dataset has been “exhausted”, sustained pressures to publish are likely to lead authors and paper mills to move on to other datasets that can be similarly exploited. The NHANES study may represent one such dataset, but it seems unlikely to be the last. This possibility highlights the need for rapid or unexpected increases in numbers of low-value manuscripts and publications to be communicated widely and acted on as quickly as possible [4], wherever these occur.

Rapidly rising numbers of articles with limited or no scientific value will clearly require targeted, co-ordinated and sustained efforts by funders, institutions, publishers and individual research teams (Table 1). As part of these efforts, rare publications such as that of Suchak and colleagues are critically important. These studies provide timely, detailed descriptions of concerning publication trends, and raise awareness of opportunities to scale publications in fields offering many individual topics that can be addressed by different authors and then published across many journals over time [9]. By judiciously increasing the levels of friction within scientific publishing, we can collectively ensure that the research use and benefits of AI outweigh their possible misuse and that science fiction is only found in novels, and not the research literature.

## Abbreviations

**:**
AI: artificial intelligence
NHANES: National Health and Nutrition Examination Survey
